# Adaptive cancer therapy: can non-genetic factors become its achilles heel?

**DOI:** 10.1038/s41388-025-03582-y

**Published:** 2025-09-22

**Authors:** Gábor Valcz, Robert A. Gatenby, Beáta Újvári, Edit I. Buzás, Béla Molnár

**Affiliations:** 1 HUN-REN-SU Translational Extracellular Vesicle Research Group, Budapest, Hungary; 2 Department of Internal Medicine and Oncology, Semmelweis University, Budapest, Hungary; 3 Cancer Biology and Evolution Program, Moffitt Cancer Center, Tampa, FL USA; 4 Integrated Mathematical Oncology Department, Moffitt Cancer Center, Tampa, FL USA; 5 School of Life and Environmental Sciences, Deakin University, Waurn Ponds, VIC Australia; 6 Institute of Genetics, Cell- and Immunobiology, Semmelweis University, Budapest, Hungary; 7 HCEMM-SU Extracellular Vesicles Research Group, Budapest, Hungary

**Keywords:** Cancer therapy, Tumour heterogeneity

## Abstract

The recurrence of clinically advanced cancers is an evolutionary consequence of standard-of-care chemotherapies generally administered at maximum tolerated doses to kill as many cancer cells as possible. The inevitable appearance of resistance raises the possibility of shifting treatment goals from complete tumor eradication to long-term disease control. The latter approach is employed by adaptive therapy, which aims to inhibit the evolutionary dynamics governing the spread of resistant tumor phenotypes. Adaptive therapy changes focus from the cancer cells that are responsive to therapy to those that are resistant and ultimately govern outcome. This therapeutic approach retains a pool of sensitive cancer cells to compete with the therapy-resistant ones through dynamic dose modulation and/or timing. Thus, fluctuations of treatment-sensitive cells are used to control the resistant population and prolong tumor control with existing therapy agents. Here, we explore non-genetic mechanisms of resistance, including the protective role of the tumor stroma, the epithelial-to-mesenchymal transition, the overexpression of drug efflux pumps, and the extracellular vesicle-mediated transfer of them. These mechanisms can increase the size of the resistant population at the expense of the sensitive one, reducing the ability of adaptive therapy to force tumor evolution into controllable cycles.

## Introduction

**Fig. 1:**
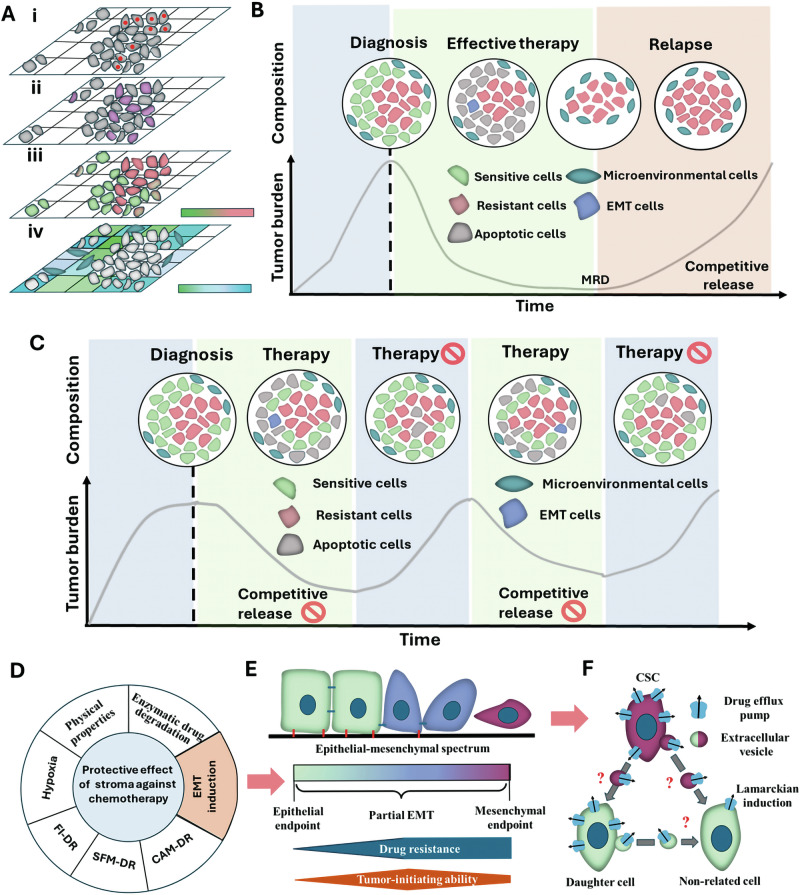
Intratumoral heterogeneity, the dynamics of maximum tolerated dose (MTD) treatment and adaptive therapy (AT), and the non-genetic mechanisms of chemoresistance. **A** Different levels of spatial heterogeneity associated with drug resistance. (i) resistance mutations in a clone (indicated by red dots), (ii) epigenetically regulated resistance (the resistant cells are marked in magenta), (iii) phenotypic resistance defined by genetic and epigenetic factors (scale: from resistant (red) to susceptible (green), (iv) microenvironmental resistance (scale: from sensitive (green) to resistant (blue). **B** The fixed maximum tolerated doses (MTD) therapies remove the sensitive cells (marked in green) by inducing apoptosis in them (gray cells), and then the resistant cells (red cells) remain as a measurable residual disease (MRD). If the cells of MRD do not die due to random effects, they develop into clinical relapse through competitive release. **C** During adaptive therapy (AT), a stable tumor volume can be maintained, and competitive release can be prevented by temporarily suspending the treatment and choosing the minimal effective dose. During therapy breaks, the gray color represents the loss of resistant cells due to intratumoral competition with sensitive cells. **D** Factors that trigger the protective effect of the tumor stroma (EMT: epithelial-to-mesenchymal transition; CAM-DR: cell adhesion-mediated drug resistance; SFM-DR: soluble factor-mediated drug resistance; FI-DR: force-induced drug resistance). **E** The epithelial-mesenchymal phenotypic spectrum (light green marked the full epithelial features (marked in the spectrum as epithelial endpoint)), and burgundy indicates the completely mesenchymal phenotype (marked in the spectrum as mesenchymal endpoint). The drug resistance and increased tumor-initiating capacity may appear during partial EMT (after *Shibue & Weinberg* [43]). **F** Extracellular vesicle (EV)-based horizontal transfer of the drug efflux pump-associated resistance properties of CSCs presumably occurs between cells at different hierarchical levels (e.g., between CSCs (burgundy) and daughter cells (green) or between two daughter cells with different lineages). The expression of drug pumps is also enhanced by Lamarckian induction.

The diagnosis of cancer is frequently perceived as a fundamental threat to existence, triggering a strong desire to completely and quickly eradicate the disease as a general goal of cancer treatment [1]. This “hit hard and fast” urge is partially satisfied by protocol-fixed, systemically administered cytotoxic therapies. These therapies are typically administered at or near the maximum tolerated dose (MTD), often within the shortest possible time (“maximum dose density”), to induce lethal toxicity in as many cancer cells as possible [2]. Although this strategy often results in significant tumor response, these aggressive therapies inevitably select for resistant phenotypes within the large, heterogeneous cancer population leading to treatment failure and tumor progression (intratumoral heterogeneity illustrated in Fig. 1A) [1, 3]. In essence, even the most effective current therapies eventually fail because cancer functions as an independent eco-evolutionary system, capable of adapting to potentially lethal therapeutic interventions by developing and disseminating dynamic, adaptive strategies [3, 4]. Cells that possess the molecular mechanisms necessary for overcoming therapeutic challenges form surviving foci called “minimal residual disease” (MRD), which is usually undetectable by clinical tests [3, 5]. Paradoxically, MTD treatment only worsens the scenario by accelerating the elimination of sensitive cells. So, it can “release” the existing resistant cells from competition-caused suppression resulting in overgrowth of minimal residual disease (Fig. 1B) [6]. Thus, the dominance and rapid expansion of resistance are a consequence of the evolutionary mechanism known as competitive release [1, 7, 8].

With the recognition of the evolutionary limitations of traditional maximum dose treatments, researchers started focusing on therapies that can steer tumor evolution to provide greater patient benefit [6, 7, 9]. Adaptive therapy (AT) was one of the first attempts at clinical handling of key evolutionary forces causing drug resistance. AT assumes resistance strategies in large cancer populations are inevitable. However, the resistant populations are initially quite small and the proliferation of those populations to become clinically significant is controlled by evolutionary dynamics that can be controlled by integrating Darwinian principles into treatment design [2, 10]. This resistance management strategy exploits the competitive interactions of dominant, drug-sensitive, and less dominant, drug-resistant subclones with repeated on-and-off cycles of the treatment (Fig. 1C) [11]. The hypothetical basis of competition is that the costs of the synthesis and operation of the resistance-related molecular machinery distract the resources needed for cell proliferation [1, 4, 12]. As a result, the resistant cells bear the energetic costs of resistance, but this provides no benefit when the therapy is temporarily suspended. This reverses the fitness relations. That is, the resistant cells are fitter than their sensitive competitors in the presence of the therapeutic agents, but the reverse is present in the absence of treatment [1, 9]. Thus, in AT, when a significant tumor response is observed, therapy is discontinued. Although the tumor population will grow in the absence of treatment, the fitness benefit of the treatment-sensitive cells allows them to proliferate at the expense of the resistant cells [1, 13]. Once the tumor burden returns to its pretreatment size, the therapy recommences, but the dominant population remains sensitive to the treatment, and the observed response is identical to the first treatment application [14]. In Table 1, we present illustrative examples from both preclinical and clinical studies investigating adaptive therapy. As can be seen, in the case of AT, it is crucial to personalize the treatment, in contrast to other on/off treatment strategies that are not tailored to the individual patient. Such a treatment strategy is “intermittent therapy”, where treatment cycling is preceded by a 7-8-month induction period during which therapy is applied at MTD, and cycled therapy is implemented only if the induction period significantly reduces the tumor burden. Under this schedule, the on/off treatment was generally found to be no better than standard of care dosing [15, 16]. In intermittent therapy, the induction period, by eliminating or greatly reducing the treatment-sensitive population, defeated the key dynamics of AT in which cycling of the treatment-sensitive population is used to control the resistant population [1]. Computer simulations demonstrated that the treatment protocol used in intermittent therapy would be no better than standard-of-care, a conclusion that is consistent with the outcomes observed in the clinical studies [14–16]. The difference in outcomes of standard-of-care, intermittent therapy, and AT highlights the necessity of formal integration of evolutionary principles in trial design and the value of mathematical modeling and computer simulations to anticipate outcomes.

**Table 1:** From preclinical models towards clinical practice: examples of completed and ongoing trials of adaptive therapy.

Type of cancer	Short summary	Ref
Breast cancer	A breast cancer xenograft model was employed to compare adaptive therapy (AT) approaches—namely, dose reduction and dose skipping—with conventional treatment. The minimum effective dose AT approach achieved stable tumor control and prolonged progression-free survival by adjusting paclitaxel based on tumor response. Tumor burden was monitored using MRI.	[9]
Melanoma	The study demonstrated that transcriptional heterogeneity in melanoma impacts the effectiveness of BRAF inhibitor therapy. Moreover, tailoring AT schedules to modulate this heterogeneity can postpone the development of resistance. AT resulted in significantly better tumor control in the xenograft mouse melanoma models than continuous and fixed-schedule treatments.	[68]
Prostate cancer	In this clinical pilot study (NCT02415621), patients with mCRPC received AT using abiraterone, which was paused when PSA dropped below 50% of baseline and resumed when PSA rebounded to pre-treatment values. Compared to MTD therapy (*n* = 16 patients), AT (*n* = 11) extended median time to radiographic progression from 13.2 to 24.0 months. The average cumulative dose of abiraterone under the AT was just 47% of that used in standard-of-care regimens.	[14]
Prostate cancer	In a pilot study (NCT02415621), evolution-based mathematical models guided abiraterone dosing in mCRPC. Patients receiving AT (*n* = 17) were compared to standard-of-care (*n* = 16), with PSA used to track tumor burden. Compared to standard therapy, AT significantly extended median time to progression (14.3 vs. 33.5 months) and overall survival (31.3 vs. 58.5 months).	[69]
Prostate cancer	Given the anticipated clinical benefits, a phase II, randomized, controlled trial (ANZadapt, NCT05393791) was initiated to assess the efficacy of abiraterone- and enzalutamide-based AT compared to standard treatment in 168 patients with mCRPC. The trial is ongoing, and the outcomes are pending.	[70]
Ovarian cancer	Mice were injected with carboplatin-sensitive and -resistant ovarian cancer cells in varying ratios. At an 80:20 sensitive-to-resistant ratio, AT achieved sustained tumor control, with median survival not reached due to too few deaths, while standard therapy resulted in a median survival of 11.25 weeks. Furthermore, the authors examine the potential of patient-derived cfDNA as a tool for monitoring the size of the resistant tumor subpopulation.	[17]
Ovarian cancer	The ongoing phase II randomized controlled clinical trial, Adaptive ChemoTherapy in Ovarian Cancer (ACTOv, NCT05080556) aims to enroll 80 patients. Within the AT framework, carboplatin dosing will be adjusted based on fluctuations in the serum biomarker CA125, which serves as a surrogate indicator of overall tumor burden.	[18]

*AT* adaptive therapy, *cfDNA* cell-free DNA, *mCRPC* metastatic castration-resistant prostate cancer, *MRI* magnetic resonance imaging, *MTD* maximum tolerated dose, *PSA* prostate-specific antigen.

AT is a treatment response feedback-based approach that requires accurate, real-time tumor burden monitoring. Dynamic changes in tumor size can be longitudinally monitored through liquid biopsies, which allow tracking of specific tumor markers such as CA125 (for ovarian cancer) [17, 18], PSA (for prostate cancer) [14], and tumor-derived cell-free DNA (cfDNA) [17]. cfDNA reflects the amount of the tumor burden [19, 20]. Furthermore, the specific pattern of copy number alterations present in an emerging, resistant population can be identified in cfDNA, allowing the size of this population to be estimated during AT [17].

Standard-of-care imaging methods such as MRI and CT primarily reflect anatomical properties and are typically limited to one- or two-dimensional tumor size measurements [21, 22]. However, quantitative image analysis from these examinations (radiomics) enables the high-throughput extraction of spatially resolved features, such as texture and intensity, that reflect underlying heterogeneity within tumors. Radiomics data can be used to identify regionally distinct tumor habitats, which may harbor cancer cell populations with different adaptive phenotypes and treatment resistance [22, 23]. Given that radiomics data originate from non-invasive and non-destructive imaging modalities, they offer the opportunity to longitudinally monitor tumor evolution during the cycles of AT. However, results from such studies are still awaited.

## Non-genetic/epigenetic components in therapy resistance

Fixed-schedule MTD therapies have the potential to achieve a complete cure if they eliminate all tumor cells before resistance emerges and spreads. In such cases, the tumor can be considered homogeneously sensitive, with no inherited or de novo emerging resistant phenotype in the size that is sufficiently large for evolution rescue [2, 7]. In contrast with MTD treatment, AT can be most effective when the frequency of resistance exceeds a minimum threshold of cell number which MTD therapy could not eliminate, but beyond that threshold, the resistant population is as small as possible [24].

Generally, in advanced cancers, complete sensitivity is rarely found. Instead, the underlying genetic and phenotypic variability ensures some degree of resistance, which is present even before the commencement of therapy [7]. Resistance mutations (or other genetic alterations with adaptive effects) present in the population allow rapid adjustment of the transcriptional and metabolic programs to therapeutic challenges [5]. Indisputably, genetic evolution is important in the overgrowth of resistant subclones. Despite this, therapies which target only the genetic events that cause resistance, often fail. Furthermore, therapeutic resistance without an identifiable genetic cause is increasingly recognized in various cancers [5, 25]. From this, we can conclude that, in addition to genetic alterations, or independently of them, non-genetic evolution is likely a significant factor contributing to drug resistance in cancer. The non-genetic resistance can involve epigenetic modifications (changes in cell phenotype caused by any means other than changes in the underlying DNA sequence) [25]. Through aberrant epigenetic regulation, cancer cells leverage the accessibility of most phenotypic properties encoded in the human genome. This is an extraordinary ability to develop adaptive strategies even against the most effective current therapies [7]. So, this state of resistance does not arise through the genetic selection of pre-existing traits; instead, these changes are triggered by a direct response to the adverse effect of the environment (see Lamarckian induction below) [25]. The epigenetic states selected by the treatment can be inherited to a certain degree by the offspring cells (transgenerational epigenetic inheritance), so a trait that provides an adaptive advantage may persist in the population of cells for a long time [26–28]. The tumor microenvironment confers additional survival advantage in the presence of the drug. It may induce a more resistant phenotype by tumor-stroma crosstalk, or by restricting drug accessibility through its physical (mechanical) properties (as not genetic and not epigenetic effect) [29, 30].

Resistance against treatments remains a major challenge in all cancer types and therapeutic modes, including targeted therapies, immunotherapy, and chemotherapy [31]. Here, we explore non-genetic resistance mechanisms, highlighting examples from chemotherapy-based studies. These main, non-genetic mechanisms include: (i) the protective effect of the tumor microenvironment, (ii) induction of epithelial-to-mesenchymal transition (EMT), and (iii) the overexpression and spreading of drug efflux pumps. Understanding these fast resistance processes can be particularly important in the case of AT when they increase the size of the resistant cell population at the expense of the sensitive one. Thus, they reduce the controllability of tumor growth, and through the development of multidrug resistance (MDR), eventually lead to a malignant relapse.

## Protective role of tumor microenvironment

So far, most experiments have mainly focused on the intrinsic chemotherapy resistance of cancer cells. Drug sensitivity observed in in vitro experiments frequently fails to correlate with the in vivo therapeutic response of tumors from which the studied cells are derived [32]. This highlights an important difference between in vitro experiments focusing on single cell response and the in vivo state of cancer cells where they are part of a complex ecosystem. This ecosystem includes other tumor cells, forming group phenotypic compositions that can promote tumor progression through selection for function [33, 34]. It also contains various components of the microenvironment, such as non-tumor host cells (e.g., cancer-associated fibroblasts, endothelial cells of the abnormal vasculature, stem-, and immune cells) and the extracellular matrix (ECM) secreted by them [35]. The microenvironmental architecture of tumors is subject to spatial and temporal changes in blood flow that may rapidly change microenvironmental conditions, size, and spatial heterogeneity, strongly influencing the evolutionary dynamics of cancers. On the one hand, the cancer ecosystem contains elements that may contribute to the extinction of the cancer population during treatment. As an example, stromal cells are known to act as competitors for resources and space with the tumor cells [36]. On the other hand, the microenvironment plays a tumor-protective role, which is further enhanced by the stromal stress caused by chemotherapy [30]. The physical (mechanical) and biological properties of the tumor stroma result in mechanisms of environment-mediated drug resistance (EMDR) [29, 37, 38]. EMDR encompasses cell adhesion-mediated drug resistance (CAM-DR), where tumor cell integrins adhere to stromal fibroblasts or ECM components, triggering survival signals. Furthermore, it also includes soluble factor-mediated drug resistance (SFM-DR), which involves the uptake of extracellularly secreted “public goods” that promote proliferation, invasion, and survival, fostering a therapy-resistant phenotype [29]. The effects of these juxtacrine and sophisticated paracrine crosstalks are supplemented by other factors such as interstitial fluid pressure, mechanical forces, composition, and stiffness of the ECM (force-induced drug resistance), hypoxic condition due to decreased blood flow, and drug degradation caused by stromal enzymes [30, 35, 39] (Fig. 1/D). The combined effect of these factors influences the availability and action of drugs. Furthermore, some microenvironmental signs can shift the cancer cell phenotype towards a stem-like-, and/or a poorly proliferative form (see below) [30, 35, 38, 39]. Since chemotherapeutic agents are mainly designed against highly proliferating cells (e.g., inducing apoptosis through DNA damage or inhibiting cell cycle progression) [35], these stem-like and/or poorly proliferating cells will be less sensitive to the treatment.

This complexity of resistance strategies affecting in situ sensitivity of cancer cells is not binary (i.e., the cell either dies, if it is sensitive, or survives if it is resistant). Rather, the sensitivity exists along a spectrum that is determined by the properties of both the cancer cells and their microenvironment (Fig. 1A). Overall, the tumor microenvironment may provide refuges that help tumor cells survive ordinarily lethal toxic insults, irrespective of their initial sensitivity to the drug [29, 31]. Therefore, the cause of the therapeutic failure must be found in the complex interplay between stromal cells, cancer cells, and anticancer therapies. Consequently, new therapeutic interventions may combine anticancer and antistromal strategies. This latter includes enabling the diffusion of therapeutic agents by increasing the ECM permeability, inhibiting molecules expressed/secreted by stromal cells, and limiting epithelial-stromal interactions (discussed by Valkenburg et al. [30]). As can be seen, the potential goal of these treatments would not be to maximize tumor cell elimination directly but to enhance AT’s effectiveness to achieve improved control over tumor burden [31].

## Epithelial-to-mesenchymal transition (EMT)

EMT is a key cellular program during embryonic tissue morphogenesis and wound healing in which epithelial cells lose their apical-basal polarity and cell-cell (and cell-basal membrane) connections to transition toward a spindle-shaped, mesenchymal phenotype. The non-genetic nature of EMT is proven by its reversibility (see mesenchymal-to-epithelial transition (MET)), and the mapped relevant epigenetic regulation [40, 41]. In cancer, the reprogramming event of EMT is aberrantly activated, leading not only to the acquisition of enhanced tumor-initiating and metastatic potential but also to increased resistance to various therapeutic assaults (Fig. 1E) [40, 42]. This complex resistance can be attributed to the EMT-associated acquisition of stem cell-like characteristics. Therefore, these cells are often defined as cancer stem cells (CSCs) characterized by heightened survival capabilities and a unique ability to generate complex neoplastic tissues [43]. CSC therapeutic resistance is the result of increased expression of drug efflux pumps, prevention- and enhanced repair capability of DNA damage, aldehyde dehydrogenase activity, augmented autophagy, and overactivation of survival-promoting signaling pathways (e.g., Wnt, Notch, and Hedgehog) [44].

EMT is rarely complete, instead, quasi-mesenchymal phenotypes with hybrid properties develop along the epithelial-mesenchymal spectrum (Fig. 1E). The EMT is induced by hypoxia, innate and adaptive immune responses, paracrine signals from the tumor environment, and treatment with antitumor drugs [45]. Here, we focus on Paclitaxel, which prolonged the progression-free survival during an AT approach in a mouse model where MDA-MB-231 breast cancer cells were orthotopically implanted in the mammary fat pad [9]. The AT strategy that gave the best results maintained the frequency of the treatment, but the doses were the minimum necessary to the response of the tumor. In this study, the initial Paclitaxel dose of AT was set at 20 mg/kg. If the tumor shrank by 20% or more, the dose was reduced by 50%. When the tumor grew by 20% or more, the dose was increased by 50% of the prior dose (standard therapy: 20 mg/kg of Paclitaxel twice a week for 2.5 weeks) [9]. Other studies described that Paclitaxel treatment induced EMT (and CSC phenotype) in the MDA-MB-231 xenograft mouse model. The Paclitaxel treatment combined with Eribulin (MET inducer) or TGF-β type I receptor kinase inhibitor, EW-7197, reduced the number of EMT events observed in MDA-MB-231 cells in vitro and in vivo [46, 47]. Interestingly, both studies use a Paclitaxel dose of 10 mg/kg (combined with EMT inhibitors) in vivo, strongly suggesting that the EMT program can be activated with a relatively low dose of the chemotherapeutic agent. Although the comparability of these mouse models is managed with caution, we can conclude that EMT can be a significant event that reduces the effectiveness of low-dose AT. However, the use of EMT inhibitors can be a potential solution for delaying the shift of cell phenotypes in the direction of resistance.

## Drug efflux pumps, and the mechanisms of their transfer to other cells

The task of drug efflux pumps is to reduce intracellular drug/toxin accumulation to sublethal levels (they are present in normal tissues like the kidneys and intestines, where they act as a protective mechanism against toxins from the bloodstream or those ingested) [48]. In cancers, the overexpressed drug efflux pumps extrude extensive arrays of drugs that are structurally and functionally diverse (hence the term MDR), imposing severe limitations on the therapeutic efficacy. The most extensively studied MDR transporters belong to the ATP-binding cassette (ABC) transporter superfamily, including P-glycoprotein/P-gp (MDR1, or ABCB1), multidrug resistance-associated protein 1/MRP1 (or ABCC1), and ABCG2 (or BCRP) [49]. The drug efflux mediated by MDRs is ATP-dependent; e.g., the P-gp and most other membrane pumps use approximately 2 ATP for each molecule of drug expulsion. Thus, in the case of limited resources, cells divert energy from less critical processes, like proliferation, to prioritize survival [48, 50]. Due to the continuous operation of the pumps, this energy rearrangement is maintained during the treatment breaks of AT, so the resistant cells will face a proliferation disadvantage compared to the better-adapted sensitive ones.

Previously, it was thought that a small population of cells using drug efflux pumps could adapt to therapeutic pressure, leading to their expansion according to Darwinian principles (“selection and expansion” model) [51]. However, several groups have shown that Lamarckian induction is also involved in the enrichment of the resistant population of cells. The driving force behind Lamarckian adaptation is epigenetic plasticity (the ability of a cell to modify its epigenetic state in response to internal or external stimuli with a certain degree of heritability) [25]. The drug-induced Lamarckian adaptation occurs much faster than genetic evolution. As an example, the percentage of HL60 acute myeloid leukemia cells that express MRP1 increased from ~2% to 30-40% within 24-48 h during treatment with low-dose vincristine [52]. A comparable phenomenon can be assumed when at 50 min after administration of doxorubicin, the P-gp/MDR1 RNA levels showed a strong increase (3-15-fold) in pulmonary metastatic tissues of sarcoma patients [53]. Overexpression of drug efflux pumps has been associated with CSCs undergoing EMT. Whether chemotherapy-induced increased drug pump expression can occur independently of EMT, or some degree of shift in the epithelial-mesenchymal phenotype spectrum needs to occur in every case, remains to be answered.

An interesting example of the rapid spread of non-genetic resistance is the horizontal transfer of survival competences between unrelated cells via secreted extracellular vesicles (EVs). EVs are intercellular communicators whose cargo (e.g., bioactive proteins, -lipids, and -nucleic acids) is protected by a lipid bilayer from the degrading effect of the extracellular space [54, 55]. Recently, the biomolecular corona formed around the surface of EVs was shown to represent significant external EV cargo [56, 57]. EVs play a primary role throughout the oncogenic process from tumor initiation to metastasis formation, via the development of resistance. The latter process may involve multifactorial mechanisms that participate in evading drug-induced cell death, e.g., removing the drug from the cytoplasm through packaging it into EVs, conveying antiapoptotic molecules between cancer cells, and transferring signals that condition stromal cells to promote drug resistance. EVs deliver a complex package of molecular cargo, and these regulators collectively shape the adaptive properties of the recipient cell. For instance, non-coding RNAs (i.e., miRNAs, lncRNAs, and circRNAs) modulate gene expression in target cells toward a resistant phenotype, e.g., by promoting EMT, autophagy, the induction of the CSC state, and by influencing DNA repair pathways [58]. Here, we focus on EVs’ ability to deliver drug efflux pumps and mRNAs (whose product is the pump protein) from resistant cells to their drug-sensitive counterparts. Functioning drug-efflux pumps, such as P-gp, MRP1, and ABCG2 were detected in EVs [55]. The drug efflux pump transfer involves the budding of donor cell membrane sections containing integrated pump proteins, resulting in EV biogenesis. The EV-conveyed efflux pump proteins (e.g., P-gp) are transferred to the recipient cell membrane, where they can function and induce MDR phenotype [59]. Furthermore, small EVs can promote drug resistance in recipient cells by transferring P-gp and P-gp mRNA [60, 61]. Hypothetically, the EV-based horizontal transfer of drug efflux pumps could preserve a part of genetic diversity during treatment, because with the help of it, therapy selects for the same phenotype in independent lineages (Fig. 1/F).

A potential opportunity to circumvent cancer cell chemoresistance is the simultaneous administration of chemotherapeutic agents and efflux pump substrates (fake drugs that act as competing ligands) increasing the actual drug’s intracellular accumulation. The dose limitations of these substrates limit the viability of these approaches. For example, verapamil, a P-gp substrate, requires micromolar concentrations (~10 µM, in vitro) that are not achievable in clinical settings (the maximum concentrations observed in clinical trials were 0.5 µM) [10]. Another characteristic of these substrates is that they can maximize the cost of resistance for the cell by increasing the ATP-dependent efflux activity of drug efflux pumps [7]. This effect is more pronounced in resource-limited environments. 2-deoxyglucose (a non-metabolizable glucose analog) treatment results in sub-physiological glucose concentrations. When applied together with 2-deoxyglucose, verapamil reduced the ability of doxorubicin-resistant cells to proliferate in vitro. Computational models based on this in vitro experiment showed a 2- to 3-fold increase in progression-free survival (PFS) of hypothetical patients using AT (compared to doxorubicin MTD treatment). The PFS increased 4 to 10-fold (depending on the size of the chemoresistant subpopulation) when AT was supplemented with verapamil and 2-deoxyglucose [10].

## Concluding remarks

Although the first studies on chemotherapy already revealed the temporary nature of the therapeutic effect (e.g., Sidney Farber et al. 1948 [62]), the underlying reason for therapeutic resistance became clear only when cancer was viewed within an evolutionary framework. This paradigm shift primarily began with John Cairns’ pioneering work in 1975 [63], which described that mutant tumor cell selection operates through mechanisms similar to classical Darwinian evolution and was later expanded by Peter Nowell’s clonal evolution model in 1976 [64]. The evolutionary view of cancer is not widely accepted. The current standard of care practice continues to use the highest possible doses to kill the maximum number of cancer cells – an approach that is usually evolutionarily unwise. The concept of MTD is so deeply integrated into drug research that one of the goals of phase 1 trials is explicitly determining the maximum dose toxicity that patients can tolerate, which is subsequently used in all following investigations [7]. Consequently, there is limited data on the biological processes occurring at low-dose treatments. The research aspirations in cancer treatment are still centered on discovering “magic bullets”, that is drugs capable of eradicating all cancer cells without harming healthy ones, ultimately leading to a cure for cancer [1, 65]. As discussed here, the evolutionary rescue of the tumor prevents the chemotherapeutic agents used at maximum tolerated doses from acting as magic bullets. In a palliative clinical setting, as in most metastatic cancers-which are almost always fatal-pursuing MTD treatment is not only futile but also imposes significant toxicity on the patient [50, 66]. AT seems to have the benefit of prolonging tumor control while using less drug to reduce toxicity [4, 9, 17, 66]. Importantly, AT can maintain tumor control as long as the sensitive population is large enough to competitively inhibit the growth of resistant cells. Thus, the sensitive-to-resistant phenotypic changes greatly influence the effectiveness of AT. The phenomena described here rapidly alter the initial clonal composition toward a resistant phenotype (even within hours after therapy application), thereby reducing the population size that can be controlled therapeutically. Although, based on preclinical studies and computational models, this shift can be pharmacologically slowed. It is important to note that most experiments designed to prevent the development of resistant phenotypes generally focus on single agents, or their combinations with chemotherapeutic agents administered at MTD. Further investigations are needed to clarify how the spatial and temporal course of these processes is influenced by modulated timing and treatment dosage and whether AT exerts a broader range of selective pressure on tumor cells. Furthermore, with adjunct therapies targeting processes that result in sensitive-to-resistant phenotypic changes, AT can access more cells which are sensitive, so may be more effective than chemotherapy alone. Integrating advanced artificial intelligence-driven predictive models into AT protocols could further enhance the precision of treatment [67], with additional refinement achieved by incorporating sensitive-to-resistant phenotypic transitions into the predictive framework. Thus, AT may achieve a more stable disease control with moderate reduction of sensitive population, and more effectively guiding the tumor to the desired evolutionary state.
